# Indigenous land management as primary health care: qualitative analysis from the Interplay research project in remote Australia

**DOI:** 10.1186/s12913-018-3764-8

**Published:** 2018-12-12

**Authors:** Rosalie Schultz, Tammy Abbott, Jessica Yamaguchi, Sheree Cairney

**Affiliations:** 1 Centre for Remote Health, Flinders University, PO Box 4066, Alice Springs, NT 0871 Australia; 2 Ninti One Ltd, PO Box 3971, Alice Springs, Australia; 3 Information and Evaluation Branch, Department of the Prime Minister and Cabinet, PO Box 6500, Canberra, ACT 2600 Australia

**Keywords:** Qualitative research, Focus groups, Interviews, Primary health care, Indigenous Australians, Land management, Conservation of natural resources, Employment, Community

## Abstract

**Background:**

For Indigenous Australians, health transcends the absence of disease, and includes the health and wellbeing of their community and Country: their whole physical, cultural and spiritual environment. Stronger relationships with Country and greater involvement in cultural practices enhance the wellbeing of Indigenous Australians, and those in more remote regions have greater access to their Country and higher levels of wellbeing. However this does not translate into improvements in clinical indicators, and Indigenous Australians in more remote regions suffer higher levels of morbidity and mortality than Indigenous people in non-remote areas, and other Australians.

The Interplay research project aimed to explore how Indigenous Australians in remote regions experience high levels of wellbeing despite poor health statistics, and how services could more effectively enhance both health and wellbeing.

**Methods:**

Indigenous Australians in remote regions, together with researchers and government representatives developed a wellbeing framework, comprising government and community priorities: education, employment and health, and community, culture and empowerment respectively. To explore these priorities Indigenous community researchers recruited participants from diverse Indigenous organizations, including Indigenous land management, art, business development, education, employment, health and municipal services. Fourteen focus groups and seven interviews, involving 75 Indigenous and ten non-Indigenous service providers and users were conducted. These were recorded, transcribed and analyzed, using thematic analysis, based on the wellbeing framework.

**Results:**

Research participants highlighted Indigenous land management as a source of wellbeing, through strengthened identity and empowerment, access to traditional food sources, enjoyable physical activity, and escape from communities where high levels of alcohol are consumed. Participants described how collaboration and partnerships between services, and recognition of Indigenous languages could enhance wellbeing, while competition between services undermines wellbeing. Indigenous land management programs work across different sectors and promote collaboration between services, serving as a source of comprehensive primary health care.

**Conclusions:**

Developing primary health care to reflect distinctive health needs of Indigenous Australians will enhance their health and wellbeing, which includes their communities and Country. Indigenous land management consolidates aspects of comprehensive primary health care, providing both clinical benefits and wellbeing, and can provide a focus for service collaboration.

## Background

The health of Indigenous Australians is poor compared with that of other Australians and has been so since data were first collected on Indigenous Australians in the 1960s [1]. Indigenous peoples throughout the world, including in Australia, experience poorer health than the dominant peoples in their countries as a result of colonization, appropriation of peoples’ lands and continuing discrimination [2]. Life expectancy, infant mortality, low birthweight, and social determinants of health including education, employment, and incomes for Indigenous Australians are considerably worse than for other Australians, and the differentials are more marked than in comparable countries, such as New Zealand, Canada and USA [3].

Australian Indigenous people are diverse, being contemporary representatives of over 250 language groups throughout a vast nation, whose initial contacts with non-Indigenous colonisation stretched from the late 1700s until the Pintupi people lost their independence from settler society in the mid-1980s [4, 5]. Many socio-economic and health indicators are worse for Indigenous people in more remote regions, particularly literacy, numeracy, income, employment status, many disease risk factors, and mortality [6]. The association between remote residence and poorer health is so strong that some researchers suggest facilitating movement of Indigenous people to larger towns to improve their health [7].

However, the concept of remoteness is not meaningful for many Indigenous people, especially those who remain on the land their communities have occupied over thousands of generations [8]. Australian measures of remoteness were developed to assist in equitable distribution of government services without reference to Indigenous people [9]. For many Indigenous Australians remoteness reflects presence in their own Country, their spiritual and physical home, a place of fulfilment, meaning and identity [8]. “Country” in this sense, and throughout this article, is defined by Indigenous people, and includes land, sea, sky, rivers, sites, seasons, plants and animals; place of heritage, belonging and spirituality [10].

Indigenous Australians in remote regions describe higher levels of wellbeing and life-satisfaction than those in non-remote regions despite poorer health statistics [11]. For example, a measure of wellbeing that is used in Australia is overall life-satisfaction, based on a single question of how well people feel their life is currently going. People respond based on their own goals, perceptions and values, enabling comparisons across time, and in different cultural, age and gender groups [12]. In remote regions of Australia, Indigenous people report mean life satisfaction of 7.6; compared with 7.2 for Indigenous people in urban regions and 7.6 for the total Australian population [13, 14].

Enhancing wellbeing is a function of primary health care, together with responding to individual and community needs, and promoting social justice and leadership for better health. Primary health care includes advocacy for economic, social and community development, and health promotion, to complement clinical services. Indigenous people’s specific needs require attention to ensure the effectiveness of primary health care services [15, 16].

Indigenous Australians conceive of health holistically, as an attribute of individuals and their community, which contrasts with narrower individual and clinical understandings of health. Indigenous people’s health includes social, emotional and cultural factors, and is a means to wellbeing rather than a goal in itself [17, 18]. Recognizing their distinctive health service needs, Indigenous Australians and their supporters have established community controlled health care services since the 1970s. A range of state and national government sources fund these services, but their dependence on government funding limits their capacity to provide genuinely community controlled services. For example, undertakings such as breakfast for undernourished school children, literacy classes, or transport for bereaved people to attend funerals are not supported by funding arrangements, even though clinical improvements have been demonstrated through such approaches [19]. Despite efforts to achieve Indigenous control of the services, there is a focus on defined clinical activity to account for government funding [20].

The disease focus of primary health care for Indigenous Australians emphasizes monitoring and surveillance of conditions of greatest burden, focusing on people and conditions of highest risk, and prioritizing interventions with evidence of greatest clinical benefit [21]. This epidemiological, disease and risk focused approach is explicated in funding arrangements, which require health services to report their clinical performance indicators to government funding agencies, including behaviours and risk factors such as prevalence of smoking, alcohol use, obesity and diabetes [22].

### Indigenous land management

Throughout Australia Indigenous land management (ILM) is increasingly being recognized for its benefits across many sectors, including health. ILM involves employment of Indigenous people to manage lands and seas, using both customary and modern techniques. Aims include harvesting of bush foods; monitoring and protection of threatened species; revegetation; control of fires, weeds and feral animals; and art and craft work. ILM depends on Indigenous people’s knowledge and skills, including languages and cultural expertise. Colloquially ILM is known as caring for Country [23]. Since employment rates of Indigenous people in remote Australia are approximately 30%, ILM is an innovative approach to complex disadvantage and disempowerment [24].

Indigenous Australians have managed Australia’s ecosystems over millennia, and on-going human involvement appears critical for ecosystem function. Recognition of the role of Indigenous people in land management has led to the establishment of Indigenous Protected Areas (IPAs) where Indigenous people are supported to undertake conservation activities on their traditional lands in accordance with Australia’s international conservation commitments such as to the IUCN (International Union for the Conservation of Nature). IPAs now comprise almost half of Australia’s nature reserves [25] so Indigenous people’s knowledge and skills are needed for Australia to maintain its conservation estate and meet international environmental commitments [26].

The wellbeing that people experience from involvement in ILM led to Indigenous community leaders asking for research into relationships between their involvement in ILM and clinical indicators. This showed that greater participation in ILM was associated with increased physical activity, better diet, and lower body weight, blood pressure, blood sugar and cholesterol [27].

The Interplay research project explored wellbeing of Indigenous Australians in remote regions who experience high levels of wellbeing despite poor health statistics. In this article, we investigated the role of ILM in wellbeing, through thematic analysis of focus groups and interviews. The Indigenous peoples of Australia comprise both Aboriginal and Torres Strait Islander peoples. For consistency with the term Indigenous land management (ILM) and international implications, we have used the capitalized term “Indigenous people” in this article for Aboriginal and Torres Strait Islander Australians. Without capitalization, “indigenous” refers to indigenous peoples worldwide [28].

## Methods: The Interplay project

### Research design and methodology

The Interplay project was a wide-ranging exploration of wellbeing for Indigenous people in remote regions of Australia, carried out through the Cooperative Research Centre for Remote Economic Participation [29]. The project brought together Indigenous community members, researchers and government agencies who are responsible for funding decisions. Qualitative methods were used to increase researchers’ understanding of wellbeing through exchange of experiences, ideas and values, and particularly to explore cross-cultural differences in understanding of wellbeing [30]. The focus of the Interplay project was on positive experiences and stories, to build a policy approach based on Indigenous people’s strengths, and provide an alternative to the negative perceptions of Indigenous people that pervade the literature and undermine Indigenous people’s wellbeing [31].

**Fig. 1:**
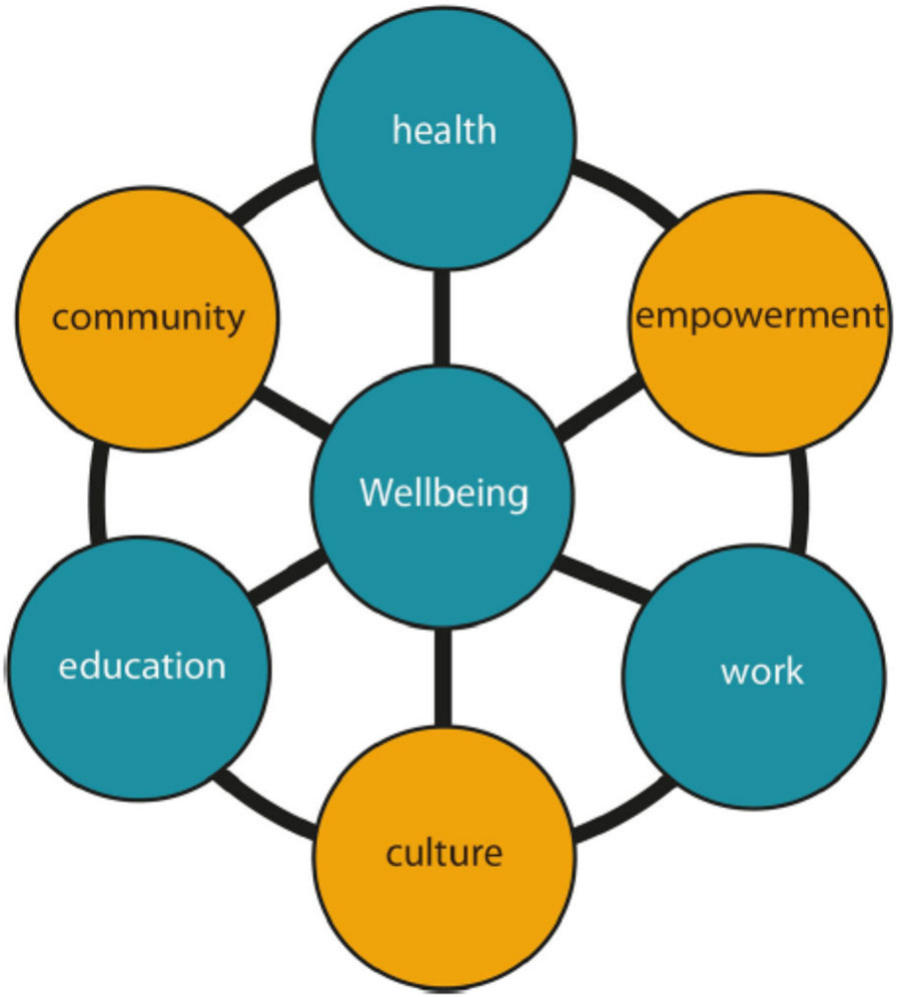
Interplay wellbeing framework, showing government priorities in blue and communities priorities in yellow

The Interplay project prioritized Indigenous people’s research interests and perspectives throughout the process, which began by developing research methodology and a wellbeing framework. This comprised wellbeing priorities for government agencies, namely education, employment and health, and for community members, namely community, culture and empowerment. The framework is shown in Fig. 1 [29].

Focus groups were chosen as the main data collection method, because they enable intercultural communication and understanding, and allow participants to share and build on one another’s ideas. Focus groups can empower participants through enabling them to guide the focus of the research, criticize services and provide solutions in a confidential setting [32]. Interviews were conducted for convenience of people who were unable to participate in the focus groups, but whose contribution was considered valuable in providing insights to wellbeing for Indigenous people in remote communities.

### Participants, sampling and data collection

Indigenous communities in the jurisdictions of Northern Territory and Western Australia who had previous research experience with the Cooperative Research Centre were invited to participate in the project. Communities were selected to achieve diversity in community geography, population size, proportion of people in the community who are Indigenous, and extent of use of Indigenous languages. Research capacity enabled four communities to participate.

Indigenous community researchers were employed to conduct research in each of these communities in 2014 and 2015. They recruited participants who could speak English through their interpersonal networks and service organizations. This approach ensured that focus group members knew one another and many worked together as service providers, so within the focus groups, participants could share and compare understandings [32]. Participants were recruited for 14 focus groups, which were facilitated by the Indigenous community researchers in English, after sharing of ideas in English and Indigenous languages, to enable participation regardless of English proficiency [33]. Notes were taken during the focus group to aid the transcribing process.

Focus groups explored participants’ views and experiences of wellbeing. Interviews followed similar format to the focus groups, conducted by Indigenous community researchers and guided by interviewees. The focus was on what works well and why. Focus groups and interviews each lasted approximately 1 h, following which participants were offered food and drink but no payment.

Information on focus group members and interviewees’ services, role, and demography is summarized in Tables 1 and 2.

**Table 1:** Research participants in focus groups by service, role, Indigenous status and gender

	Service	Participant role	Total participants	Indigenous participants	Female participants
1	Business development	Managers	2	1	0
2	Education	Managers, employees	6	6	6
3	Education	Managers, employees	12	12	9
4	Indigenous Land Management	Participants	4	4	3
5	Indigenous Land Management	Employees	4	4	1
6	Indigenous Land Management	Employees	4	4	4
7	Indigenous Land Management	Employees	7	7	0
8	Indigenous Land Management	Employees	8	8	8
9	Health	Managers, employees	4	1	1
10	Health	Employees, community members	9	9	0
11	Health	Employees	3	1	3
12	Municipal services	Managers, employees	6	6	4
13	Municipal services	Managers	2	1	1
14	Research	Employees	4	4	4
	Total		75	68	44

To maintain privacy, numbers in the Table do not correspond to letters used to identify the focus groups in the text

**Table 2:** Research participants in interviews by service, Indigenous status and gender

Service provided by interviewee	Total Interviewees	Indigenous interviewees	Female interviewees
Indigenous Art	1	1	1
Business development	1	1	0
Education	2	2	2
Indigenous Land Management	3	1	1
Total	7	5	4

### Analysis

Focus groups and interviews were audio-recorded, then transcribed, coded, interpreted and analyzed. The priorities of the wellbeing framework, shown in Fig. 1, provided themes for initial coding and inductive analysis. The importance of ILM to wellbeing which had emerged in development of the project provided a coherent central concept [34]. This enabled development of Aboriginal perspectives of health and wellbeing expressed in the focus groups and interviews to be formulated as an approach to health care services through ILM.

### Interpretative rigour

Conduct of the research through the Cooperative Research Centre for Remote Economic Participation required mutual understanding to support the on-going relationships which are the basis of the Research Centre, particularly between Indigenous and non-Indigenous researchers. This provided credibility and rigor for all aspects of the research, including transcribing, interpreting, analyzing and reporting the findings [35, 36].

### Ethics

Engagement and support from the Indigenous services and organizations involved were fundamental for the project. Northern Territory Department of Health/ Menzies School of Health Research Ethics Committee (Reference 2013–2125), and the Western Australian Aboriginal Health Ethics Committee (Reference 549) provided formal ethical approval.

## Results

### Wellbeing through indigenous land management

Indigenous land management (ILM) services were the main sector represented by participants in the Interplay focus groups and interviews. ILM emerged as a contributor to wellbeing through participants from many sectors including business development, education, health and municipal services.

Indigenous participants involved in ILM described how the work enhances their wellbeing, through recognizing their identity and relationships with the Country. For example:

“Makes me feel good going out on country. Looking forward to getting up for work – I want to go back and see that place again and again.” (ILM program participant: focus group A).

“My benefit is the land and the sea… It made me strong and it changed my life to be stronger.” (ILM program participant: focus group G).

Indigenous people can experience deep relationships with their Country, as if the Country is part of their family. One participant explained:

“[The Country is] in your bloodline, you know.” (ILM program participant: focus group G).

Participant’s identification of Country as a family member, meant that for him ILM or caring for Country is caring for his family.

Empowerment is a community priority of the Interplay wellbeing framework, and ILM supports empowerment through employment, a government priority:

“It’s all about empowerment, you’re empowering yourself to go to work every day.” (ILM program participant: focus group G).

### Health and education through indigenous land management

Participants in the Interplay project described how ILM provides them with direct clinical benefits. These include mental and physical health benefits from exercise and quality diet from harvest of traditional foods.

“What are the foods that they hunt for?”

“They go fishing, kangaroo, pigeon, goanna, rock wallaby.”

“Sugar bag, yep, bush honey.” (ILM program participant: focus group E).

“Tons of physical activity – the blokes work really hard – manual labour at parks and wildlife centre and in [the Indigenous Protected Area].” (Non-Indigenous interviewee, ILM program coordinator).

Complementing benefits in nutrition and physical activity, employment in ILM reduces access to alcohol and associated harms, as explained by focus group participants:

“[Our people are] getting out and away from the community and some of the men said you know ‘It’s good you know, you’re away from alcohol, you’re out on the Country.’ It’s good.” (ILM program participant: focus group A).

“Gets [our people] away from the streets and from alcohol and drugs.” (ILM program participant: focus group B).

ILM provides an opportunity for education of young people and transmission of knowledge that Indigenous people value, as described here:

“Through learning on Country, we are actually … making sure our Country is safe, our next generation of young people get educated, in both ways have a healthy life, healthy community, and healthy people…. It forms a career pathway.” (ILM program participant: focus group G).

“[Indigenous] people came up with this, started exploring the link between health and education the [Indigenous] way.” (Indigenous Education program: focus group K).

### Community wellbeing and health

Research participants described the value of ILM in supporting community health, as for example:

“The [ILM] organization’s growing because of the unity and the friends …we work together, learn together, being healthy together. Healthy workplace, healthy Rangers. [We are] …empowering our organization to be a strong, and we’ll have a sustainable future.” (ILM program participant: focus group G).

“Working on Country … is one of the most important things we have in these communities, as community and land connects everyone together, emotionally, physically, spiritually and culturally. The Country and being in charge of managing it is extremely important to people … across Indigenous communities in Australia.” (Non-Indigenous interviewee, ILM program coordinator).

Focus group participants also identified the importance of their languages to their wellbeing:

“[Indigenous] people want to talk their language … it’s like they got their language they speak all the time. Why don’t we speak our languages? …. [we] want to be recognized.” (ILM program participant: focus group B).

“They should [use] our language, that [Indigenous] language. We should be there, coz we’re the first nations of the land.” (ILM program participant: focus group B).

These findings show how ILM contributes to sustaining and promoting Indigenous languages, for the benefit of speakers, their communities, and the land management knowledge communicated through Indigenous languages.

### Interplaying services: alternative frameworks for wellbeing

ILM service providers described how their organizations provide integrated services. For example:

“… [we provide] support across the board like a wrap-around service.” (Non-Indigenous interviewee, ILM program coordinator).

Indigenous research participants described frustration at the competition between different organizations for resources. From their perspective, funding from separate government bureaucracies creates barriers to effective service provision. Competition for resources between services is particularly problematic in small communities where service providers and users may be from the same families. Research participants stated:

“It all comes back to the same thing, the funding competing for each other. It’s all in silos still. If you say interplay between education and health that’s not how it happens on the ground.” (Indigenous Research organization: focus group F).

“[It’s a problem for us] competition. We are talking about people’s lives” (ILM program participant: focus group J).

In the remote communities of the research, separation of services through government policy frameworks creates barriers to collaboration and teamwork. Participants described how even within one service sector, such as health, service providers must compete for funds. This can undermine relationships within families and communities which is counterproductive for wellbeing.

“Some of the NGOs feel threatened by us [Indigenous organization] at times. Like the Red Cross and Anglicare and Centrecare, … they all going for this Indigenous funding… So there is competition for funding.” (ILM program participant: focus group E).

Non-Indigenous participants drew attention to bureaucratic barriers to professionals providing effective services in remote Indigenous communities. Specific instances were described of Indigenous health educators and psychologists being barred from providing services outside established settings because of funding or registration restrictions.

In contrast, a collaborative or partnership approach shares Indigenous people’s values, as the interviewee here reported:

“We’ve had to look at other ways to fund from other avenues and partnerships to pull money in and taking this on like an enterprise rather than a centrally funded idea.” (Non-Indigenous interviewee, ILM program coordinator).

## Discussion

In the Interplay project, the opportunity for ILM to enhance wellbeing emerged in discussions with service providers and users in a range of sectors. Research participants described how ILM promotes wellbeing through strengthening people’s sense of identity and important relationships, empowering people, providing access to traditional foods and physical activity, limiting access to alcohol, and strengthening and promoting collaboration of community organizations.

### Indigenous land management enhances wellbeing and provides comprehensive primary health care

Participants in the Interplay project described how ILM builds on their strengths, identity and relationships. This contrasts current service provision for Indigenous people, which focuses on problems perceived in Indigenous people, such as poor health status, unemployment and lack of educational attainment. Services established on the basis of negative comparisons of Indigenous with non-Indigenous Australians contribute to negative perceptions of Indigenous peoples, pejorative stereotypes and perceptions that Indigenous Australians are intrinsically problematic [37]. In contrast, Interplay research participants described how ILM programs arise from common goals of government and Indigenous community members including improving employment and education outcomes, promoting better diet and physical activity, and reducing access to alcohol.

Misuse of alcohol by Indigenous people is a particularly challenging problem when harmful levels of alcohol consumption are a community norm, so interventions with individuals may be ineffective [38]. Because it services the community, ILM provides a strength-based intervention to reduce alcohol consumption and harm.

Negative statistics on Indigenous people’s health are pervasive. Although their use is intended to motivate health care service providers to provide quality health care for Indigenous people, they also contribute to undermining the wellbeing of Indigenous people whose self-perceptions are of pride, strength and survival [31]. More culturally attuned health services for Indigenous people could monitor their performance with indicators based on Indigenous people’s own concepts of health and wellbeing. Validated measures of participation in ILM have been developed, and these are associated with clinical indicators [39]. Measures of cultural education and practice, and valuing Indigenous law and ceremony have also been validated as indicators of wellbeing [39, 40]. These could be incorporated into the performance indicators that Indigenous health services report to the Australian government to complement current clinical performance indicators. Greater emphasis on non-clinical aspects of health service provision will drive more culturally attuned health services for Indigenous people, and recognize their distinctive health needs.

Indigenous people in remote communities have lower participation in paid employment than any other group in Australia. This is attributed to prioritization of family and community responsibilities over paid employment, and remoteness [24]. From the perspective of Indigenous people, many employment options require them to separate commitments to work from their community relationships, in exchange for monetary income. None of the participants in the Interplay project mentioned income as a benefit of employment or ILM, pointing to a low priority of financial incentives. However participation in ILM provides employment that can strengthen relationships, build cultural knowledge and skills, and enable people to remain in communities considered by government to be remote [23].

With increasing recognition of the rights of Indigenous Australians to their Country, Indigenous people now manage over half of Australia’s total land area, and over 70% of the land protected for conservation [41]. Thus ILM is of growing importance to Australia’s international commitments to biodiversity protection and sustainable development. Relationships between environmental sustainability and the wellbeing of Indigenous people, through ILM, have been under-recognised in Australia’s policy development, reflecting the separation of government departments [42].

Interplay research participants described how their languages provide a source of identity and wellbeing and their disappointment that their languages are not recognised. Lack of Indigenous language training and use of interpreters by health care service providers appears widespread [43]. Indigenous language use itself is an important determinant of health, which is promoted by ILM [44]. These finding suggest that greater recognition of the need for communication in Indigenous languages would improve health service accessibility for Indigenous Australians. Indigenous language translation or knowledge as a performance indicator for health care services for Indigenous Australian could drive increases in this critical element of health care [45].

### Intersectoral contributors to health

Indigenous experts have called for a transformation in health care for Indigenous Australians because of the unacceptable costs of current vertical, disease-focused approaches, and noted that this will require reshaping policies, reinventing organizations, and working across sectors [46].

Considering ILM as health care overcomes the tension between promoting the healthy lifestyle conceptualized by non-Indigenous Australians, which may themselves contribute to on-going colonisation, and the Indigenous disadvantage that is attributable to unhealthy behaviours [47]. ILM involvement improves lifestyle contributors to health, through providing access to traditional foods and physical activity. Indigenous knowledge maintains an holistic perspective on the country including its people, in which people’s health depends on the health of the country [48, 49]. Community gardens, like ILM, have also been identified as a source of comprehensive primary health care for Australians [50]. This suggest a broader primary health care movement towards outdoor, productive, collaborative activities may provide health benefits to complement clinical health services.

Re-conceptualising service provision based on the needs of Indigenous people and communities in remote regions could have benefits in many sectors. Interplay project participants provide and receive services through separate government sectors, reflecting decisions about resource allocation based on non-Indigenous priorities [40]. For Indigenous people these decisions can appear arbitrary. The Interplay project showed how government service priorities of education, employment and health work together with people’s priorities in community, culture and empowerment [40].

Interplay research project participants, both Indigenous and non-Indigenous, highlighted the importance of Country to the health and wellbeing of Indigenous people, which suggests the opportunity to conceptualize ILM as a health service. This emphasizes the importance of sectors other than health to people’s wellbeing, recognized since primary health care was described in the Alma Ata Declaration of 1978 [15]. ILM is of great value because it provides services in several sectors, including land management, employment, education and health.

The importance of Country for Indigenous people, and caring for Country as a source of economic development provide an alternative basis for service development. Social analysis of four ILM programs that showed a return of $96.5 million for $35.2 million investment over 6 years, or 29% per year. This analysis included benefits of skill development, work satisfaction, employment income and ability to better provide for families, which facilitate economic and community development [51]. Scaling up these interventions to enhance the wellbeing of Indigenous people throughout remote Australia would have widespread benefits.

Interplay project participants drew attention to current relationships between services based on competition and to restrictive employment practices, which appear inefficient in remote communities. Research participants’ dislike of competition reflects their values of kinship and community relationships as priorities, rather than cost-effectiveness that is imposed by government funding agencies.

Other researchers have suggested that Australian governments should encourage Indigenous people to leave remote regions, because of the challenges of providing access to services in remote regions and the impacts of limited service access on health [52]. This would not be supported by findings from the Interplay project that Indigenous people derive of health and wellbeing benefits from ILM in remote regions. Longitudinal census and social survey data also show that individual Indigenous people’s employment prospects do not improve when they leave remote communities [53, 54]. The Interplay research showed that ILM provides health and wellbeing benefits, which complement environmental and social benefits of participation in ILM. The wellbeing that Indigenous people derive from ILM may explain their attachment to their ancestral lands despite limited employment and educational opportunities in remote regions.

### Global perspectives on indigenous land management

Health for indigenous peoples globally has distinctive features, reflecting the distinctive relationships between indigenous peoples and their lands [55]. While each indigenous group is unique, close relationships with their lands are a characteristic of indigenous peoples [56]. Thus findings about the value of land management for Indigenous Australians may be relevant for health and wellbeing of other indigenous populations. Indigenous peoples’ expertise is important globally in ensuring that ecosystems are maintained to ensure sustainable development for all of humanity. Thus both lands and peoples benefit from ILM [41, 57].

### Study limitations

Indigenous people in remote regions were the focus of this research, and represent about 21% of Indigenous Australians. Nonetheless, 73% of all Indigenous Australians recognize a homeland or traditional Country, and half visit their Country at least yearly [6]. Furthermore, ILM programs have been established throughout Australia including in urban regions, following models from remote regions [58]. Key barriers to urban Indigenous people participating in ILM are limited respect and practical support for Indigenous knowledge and worldviews and limited access to lands and waters, rather than the fact that people are not in remote regions [23].

The research team was led by women, leading to potential bias towards female perspectives and participation. However there was a high representation of male participants particularly in the ILM programs.

## Conclusions

For Indigenous Australians, ILM provides opportunities for promoting both individual and community health and wellbeing through empowerment, healthier behaviours, use of Indigenous languages and knowledge transmission across generations. ILM integrates the aims of different services, including education, employment and health, enabling sectors to work together rather than in competition. Collaboration of services through ILM will enhance service productivity and aligns with worldviews of Indigenous people. ILM is unconventional as health care but contributes to the comprehensive primary health care needs of Indigenous Australians.

## Abbreviations

ILM: Indigenous Land Management
NATSISS: National Aboriginal and Torres Strait Islander Social Survey
NGO: Non-government organisation

## References

[1] Altman J, Biddle N, Hunter B (2004). Indigenous Socioeconomic Change 1971–2001: A Historical Perspective.

[2] Stephens C, Porter J, Nettleton C, Willis R (2006). Disappearing, displaced, and undervalued: a call to action for indigenous health worldwide. Lancet.

[3] Anderson I, Robson B, Connolly M, Al-Yaman F, Bjertness E, King A (2016). Indigenous and tribal peoples’ health (the lancet–Lowitja Institute global collaboration): a population study. Lancet.

[4] Kapi KD, Batty P (2006). Contact experiences in the Western Desert 1873-1984. Colliding worlds: first contact in the Western Desert 1932–1984.

[5] Dixon RM, Blake BJ. Introduction. In: Dixon R, Blake BJ, editors. Handbook of Australian languages: 1: John Benjamins Publishing Company; 1979. p. 1–26.

[6] Australian Institute of Health and Welfare (2015). The health and welfare of Australia’s Aboriginal and Torres Strait Islander peoples 2015.

[7] Zhao Y (2017). Letter to editor re: The economic benefits of eliminating indigenous health inequality in the Northern Territory. Med J Aust.

[8] Birch T (2016). Climate change, mining and traditional indigenous knowledge in Australia. Social Inclusion.

[9] Australian Bureau of Statistics (2013). Australian statistical geography standard (ASGS): volume 5 - remoteness structure July 2011.

[10] Australian Museum (2017). Glossary of indigenous Australia terms.

[11] Biddle N, Hunter B, Biddle N (2012). Improving indigenous health: are mainstream determinants sufficient?. Survey analysis for indigenous policy in Australia: social science perspectives.

[12] OECD (2017). How’s Life? 2017: Measuring wellbeing.

[13] Australian Bureau of Statistics (2016). 4714.0 - National Aboriginal and Torres Strait Islander Social Survey, 2014–15.

[14] Australian Bureau of Statistics (2015). 4159.0 - General Social Survey: Summary Results, Australia, 2014.

[15] World Health Organisation (1978). Declaration of Alma-Ata.

[16] World Health Organisation. The world health report 2008: Primary health care now more than ever. Geneva: World Health Organisation. p. 2008. http://www.who.int/whr/2008/whr08_en.pdf. Accessed 27 Feb 2018.

[17] Australian Government (2013). National Aboriginal and Torres Strait islander health plan 2013–2023.

[18] National Aboriginal Health Strategy Working Group (1989). National Aboriginal Health Strategy.

[19] Khoury P (2015). Social anthropology and its uses for policy: beyond the biomedical paradigm: the formation and development of indigenous community-controlled health organizations in Australia. Int J Health Serv.

[20] Australian Institute of Health and Welfare (2016). Aboriginal and Torres Strait Islander health organisations: Online Services Report—key results 2014–15. Aboriginal and Torres Strait Islander health services report No. 7. IHW 168.

[21] Brough M (2001). Healthy imaginations: a social history of the epidemiology of aboriginal and Torres Strait islander health. Med Anthropol.

[22] Australian Government Department of Health. Online Services Report and National Key Performance Indicators for Aboriginal and Torres Strait Islander primary health care data framework. Canberra: Department of Health; 2015. http://www.health.gov.au/indigenous-hpf. Accessed 27 Aug 2017.

[23] Hill R, Pert PL, Davies J, Robinson CJ, Walsh F, Falco-Mammone F (2013). Indigenous land Management in Australia: extent, scope, diversity, barriers and success factors.

[24] Gray M, Hunter B, Lohoar S (2012). Increasing indigenous employment rates. Issues paper no 3. Produced for closing the gap clearinghouse.

[25] Cresswell I, Murphy H (2016). Australia: State of the environment 2016: biodiversity, independent report to the Australian government minister for the environment and energy.

[26] Commonwealth of Australia (2011). The Auditor General Audit report No 14: 2011–12 Performance Audit: Indigenous Protected Areas.

[27] Burgess CP, Johnston FH, Berry HL, McDonnell J, Yibarbuk D, Gunabarra C (2009). Healthy country, healthy people: the relationship between indigenous health status and “caring for country”. Med J Aust.

[28] Cultural Diversity and Inclusivity Practice (2015). Appropriate terminology, representations and protocols of acknowledgement for aboriginal and Torres Strait islander peoples.

[29] Cairney S, Abbott T, Yamaguchi J (2015). Study protocol: the Interplay Wellbeing Framework and methodology to assess wellbeing in Aboriginal and Torres Strait Islander people in remote Australia.

[30] Petty NJ, Thomson OP, Stew G (2012). Ready for a paradigm shift? Part 1: introducing the philosophy of qualitative research. Man Ther.

[31] Bond C (2005). A culture of ill health: public health or aboriginality?. Med J Aust.

[32] Kitzinger J (1995). Qualitative research: introducing focus groups. Br Med J.

[33] Malcolm IG (2013). The ownership of aboriginal English in Australia. World Englishes.

[34] Braun V, Clarke V (2006). Using thematic analysis in psychology. Qual Res Psychol.

[35] Laycock A, Walker D, Harrison N, Brands J (2011). Researching indigenous health: a practical guide for researchers.

[36] O'Brien BC, Harris IB, Baeckman TJ, Reed DA, Cook DA (2014). Standards for reporting qualitative research: a synthesis of recommendations. Acad Med.

[37] Walter M, Kukutai T, Taylor J (2016). Data politics and indigenous representation in Australian statistics. Indigenous data sovereignty: towards an agenda.

[38] Kowal E, Paradies Y (2010). Enduring dilemmas of indigenous health. Med J Aust.

[39] Burgess C, Berry H, Gunthorpe W, Bailie R. Development and preliminary validation of the ‘Caring for Country’ questionnaire: measurement of an indigenous Australian health determinant. Int J Equity Health. 2008;7. 10.1186/1475-9276-7-26. 10.1186/1475-9276-7-26 PMC2628914 19094240

[40] Cairney S, Abbott T, Quinn S, Yamaguchi J, Wilson B, Wakerman J (2017). Interplay wellbeing framework: a collaborative methodology ‘bringing together stories and numbers’ to quantify aboriginal cultural values in remote Australia. Int J Equity Health.

[41] Garnett ST, Burgess ND, Fa JE, Fernandez-Llamazares A, Molnar Z, Robinson CJ (2018). A spatial overview of the global importance of indigenous land for conservation. Nature Sustainability.

[42] Biddle N, Swee H (2012). The relationship between wellbeing and indigenous land, language and culture in Australia. Aust Geogr.

[43] Mitchell AG, Belton S, Johnston V, Wopurruwuy G, Ralph AP. “That heart sickness”: young aboriginal People’s understanding of rheumatic fever. Med Anthropol: Cross-Cultural Studies in Health and Illness. 2018: doi: 10.1080/01459740.2018.1482549. 10.1080/01459740.2018.1482549 30067382

[44] Flood D, Rohloff P (2018). Indigenous languages and global health. Lancet Global Health.

[45] Cass A, Lowell A, Christie M, Snelling PL, Flack M, Marrnganyin B (2002). Sharing the true stories: improving communication between aboriginal patients and healthcare workers. Med J Aust.

[46] Houston S (2016). We need transformative change in aboriginal health. Med J Aust.

[47] Kowal E (2006). Moving towards the mean: dilemmas of assimilation and improvement. Moving anthropology: critical indigenous studies.

[48] Ens E, Pert P, Clarke P, Budden M, Clubb L, Doran B (2015). Indigenous biocultural knowledge in ecosystem science and management: review and insight from Australia. Biol Conserv.

[49] Kingsley JY, Townsend M, Phillips R, Aldous D (2009). “If the land is healthy ... It makes the people healthy”: the relationship between caring for country and health for the Yorta Yorta nation, Boonwurrung and Bangerang tribes. Health Place.

[50] Marsh P, Brennan S, Vandenberg M (2018). ‘It’s not therapy, it’s gardening’: community gardens as sites of comprehensive primary healthcare. Aust J Prim Health.

[51] Social Ventures Australia. Consolidated report on indigenous protected areas following social return on investment analyses: Social Ventures Australia consulting; 2016. https://www.pmc.gov.au/sites/default/files/publications/SROI-Consolidated-Report-IPA_1.pdf. Accessed 5 Oct 2018.

[52] Zhao Y, Vemuri SR, Arya D (2016). The economic benefits of eliminating indigenous health inequality in the Northern Territory. Med J Aust.

[53] Biddle N, Crawford H. The changing Aboriginal and Torres Strait Islander population: Evidence from the 2006–11 Australian Census Longitudinal Dataset. Canberra: Centre for Aboriginal Economic Policy Research; 2015. http://caepr.cass.anu.edu.au/research/publications/changing-aboriginal-and-torres-strait-islander-population-evidence-2006-11. Accessed 21 Feb 2018.

[54] Stephens B (2010). The determinants of labour force status among indigenous Australians. Aust J Labour Econ.

[55] Charlier P, Coppens Y, Malaurie J, Brun L, Kapanga M, Hoang-Opermann V (2017). A new definition of health? An open letter of autochthonous peoples and medical anthropologists to the WHO. Eur J Intern Med.

[56] World Bank (2011). Indigenous peoples policy brief: still among the poorest of the poor: policy brief 64760.

[57] United Nations General Assembly (2015). Resolution 70/1. Transforming our world: the 2030 agenda for sustainable development.

[58] Patterson T, Hunt J, Altman J, Kerins S (2012). Reconnecting with culture for future generations. People on country: vital landscapes, indigenous futures.

[59] National Health and Medical Research Council, Australian Research Council, Universities Australia. National Statement on Ethical Conduct in Human Research 2007 (updated 2018). Canberra: Commonwealth of Australia. https://nhmrc.gov.au/about-us/publications/national-statement-ethical-conduct-human-research-2007-updated-2018. Accessed 15 Nov 2018.

